# Editorial: Novel Approaches to the Treatment of Multidrug-Resistant Bacteria

**DOI:** 10.3389/fphar.2022.972935

**Published:** 2022-08-03

**Authors:** Priyia Pusparajah, Vengadesh Letchumanan, Bey-Hing Goh, Lyndy Joy McGaw

**Affiliations:** ^1^ Medical Health and Translational Research Group (MHTR), Microbiome and Bioresource Research Strength (MBRS), Jeffrey Cheah School of Medicine and Health Sciences, Monash University Malaysia, Subang Jaya, Malaysia; ^2^ Novel Bacteria and Drug Discovery Research Group (NBDD), Microbiome and Bioresource Research Strength (MBRS), Jeffrey Cheah School of Medicine and Health Sciences, Monash University Malaysia, Subang Jaya, Malaysia; ^3^ Biofunctional Molecule Exploratory (BMEX) Research Group, School of Pharmacy, Monash University Malaysia, Subang Jaya, Malaysia; ^4^ College of Pharmaceutical Sciences, Zhejiang University, Hangzhou, China; ^5^ Phytomedicine Programme, Department of Paraclinical Sciences, Faculty of Veterinary Science, University of Pretoria, Pretoria, South Africa

**Keywords:** novel approaches, bacteria, antibacterial, multidrug resistance, biofilm

Multidrug-resistant (MDR) organisms constitute a major global health crisis with antimicrobial resistance (AMR) being among the top ten global public health threats faced by humanity as declared by the World Health Organization in 2019 (WHO, 2021). Treatment failure of bacterial infections is increasingly common, as once-susceptible organisms have now developed resistance to most first-line antibiotics, resulting in greater morbidity and mortality rates (Paitan, 2018; De Oliveira et al., 2020; Loo et al., 2020; Goh et al., 2021; Kornelsen and Kumar, 2021). Additionally, multidrug resistant organisms are now not limited to nosocomial infections, but are increasingly found in the community (van Duin and Paterson, 2016). The rapid rates at which pathogens develop resistance to antibiotics coupled with the difficulty of finding effective new antibiotics has raised fears that we may be back to the pre-antibiotic era by 2050 if no new means of treating these organisms are found (Medina and Pieper, 2016; Campanini-Salinas et al., 2018; Low et al., 2021).

Mechanisms of resistance in these organisms are myriad, including altered targets of antimicrobial drugs (be they structural proteins or enzymes), and reduction of drug concentrations in the target organism by reducing entry or by active efflux, where the chromosomal coding to enable these are often transmitted from other bacteria via plasmids (Alekshun and Levy, 2007; Magiorakos et al., 2012; Wiercińska et al., 2015; Falagas et al., 2019; De Oliveira et al., 2020). Overcoming these requires novel approaches and mindsets to formulate practical and effective means of combating bacterial and other pathogens. Alternative options include focusing on different targets, such as destruction or prevention of formation of bacterial biofilms, which shield bacteria from antibiotic action within a protective matrix, as well as host-directed immunomodulation. Combinations of promising compounds with varying bioactivities- in particular from natural sources; as well as repurposing and combining existing drugs for use as antibacterials are other potential avenues for research.

This Research Topic aimed to provide a platform for experts to share research across a range of potential novel approaches to treatment of infections with MDR bacteria–spanning those related to natural drug discovery or novel applications of existing therapeutic agents. This resulted in a collection of eight articles, mostly comprising experimental research articles, reporting on innovative approaches to deal with the rising threat of bacterial MDR.

Many bacteria have the ability to produce biofilms, comprising organised congregations of bacteria adhering to each other and to biotic or abiotic surfaces while producing extracellular matrix. Such biofilms assist in evading antibiotic action by protecting bacteria within the matrix; cells within biofilms have up to 10 000-fold higher tolerance to antimicrobials (Sharma et al., 2019). In this article collection, Escobar et al. demonstrate that Bay 11–7,085, a kinase inhibitor originally identified as a potential anti-inflammatory agent, has strong potential as an anti-biofilm agent. In a similar vein, Zhang et al. report that andrographolide sulfonate, an active component of the traditional herbal medicine *Andrographis paniculata*, inhibited the growth of methicillin-resistant *Staphylococcus aureus* (MRSA) and its biofilm. The mechanisms of action were found to involve inhibition of the expression of genes such as quorum sensing (QS) system regulatory genes (agrD and sarA) and intercellular adhesion genes (icaA, icaD, and PIA), as well as downregulation of five biofilm-related metabolites among others (Zhang et al.).

Continuing with the focus on natural products, Aro et al. investigated the *in vitro* ability of the acetone leaf extract and fractions of *Psychotria capensis* to fight co-infection with organisms implicated in causing tuberculosis and helminthiasis. Parasitic infestations are known to impair the host immune system and enhance the pathogenesis of mycobacterial infections (Abate et al., 2012). The anti-inflammatory and antibacterial compound neophytadiene was noted in the most active fraction, and it was concluded that researching complementary approaches such as decreasing the incidence of helminth infestation could reduce the burden of tuberculosis, thus contributing to slowing drug resistance (Aro et al.). Liu et al. focused their investigation on alkaloids from *Macleaya cordata*, a medicinal plant used in human as well as animal health. Alkaloids, including 6-ethoxysanguinarine (6-ES), were shown to have promising activity against MRSA. The alkaloid 6-ES suppressed the hemolytic activity of a-hemolysin, alleviated inflammatory responses, and destroyed intracellular MRSA, and also demonstrated a low development of drug resistance *in vitro*. Additionally, a 6-ES-loaded thermosensitive hydrogel promoted wound healing in mice experimentally infected with MRSA. These results support 6-ES as a novel potential candidate with antibacterial, antivirulence, and host immunomodulatory activities, all useful properties in the fight against bacterial infections.

Strengthening host defences is an important approach in assisting the body to rid itself of pathogens, including those causing microbial infections. Tian et al. investigated the ability of a non-toxic polysaccharide from eggs of *Strongylocentrotus nudus*, an edible sea urchin, to protect against bacterial infection. The polysaccharide was able to protect mice from *E. coli* induced mortality in addition to reducing pulmonary inflammation and inhibiting dissemination of bacteria to organs, among other beneficial effects (Tian et al.). It was concluded that the polysaccharide strengthened innate host defences, improving the outcome of a bacterial infection, demonstrating its potential as an immunomodulator in host-directed therapies (Tian et al.).

Synergism is a useful strategy to combine two or more therapeutic agents in the hopes of attaining a more effective treatment, against which resistance will not be so quick to develop. Kussmann et al. explored the synergistic effect both *in vitro* and *in vivo* of cefazolin and fosfomycin, finding that low concentrations of fosfomycin restored the susceptibility of methicillin-resistant *Staphylococcus aureus* (MRSA) to cefazolin.

Pharmacokinetic and pharmacodynamic data are essential for determining appropriate use of antibiotics, particularly new treatments. In an article examining the pharmacodynamics of zoliflodacin against *Neisseria gonorrhoeae* in a dynamic *in vitro* Hollow Fiber Infection Model (HFIM) for gonorrhea, Jacobsson et al. concluded that for effective *N. gonorrhoeae* eradication and resistance suppression, zoliflodacin should be administered as a single large dose. Significantly, zoliflodacin resistance amplification was noted for all doses that did not eradicate the bacteria (Jacobsson et al.).

A review article in this collection highlights advantages, disadvantages and mechanisms of action of targeted therapeutic strategies in addressing AMR (Yang et al.). The authors stress the role of indiscriminate use of broad-spectrum antibiotics in accelerating the emergence of AMR, providing insights on current and future targeted medical strategies such as nanotechnology, phage therapy and CRISPR-Cas9 technology. It is anticipated that this will contribute to the design of next-generation antimicrobial agents and successful application of these in clinical use (Yang et al.).

The work highlighted provides some hope that continued efforts may provide solutions to overcoming the defences of MDR bacteria. Future research may focus on continuing bioprospecting for novel substances, and exploring newer modalities such as phage therapy and lytic antibiotics as well as obtaining a deeper understanding of the pathways involved in MDR mechanisms (e.g., regulatory mechanisms of efflux pumps) in order to engineer targeted drugs.
